# Dispositional Affect as a Moderator in the Relationship Between Role Conflict and Exposure to Bullying Behaviors

**DOI:** 10.3389/fpsyg.2019.00044

**Published:** 2019-01-24

**Authors:** Iselin Reknes, Ståle Valvatne Einarsen, Johannes Gjerstad, Morten Birkeland Nielsen

**Affiliations:** ^1^Department of Psychosocial Science, University of Bergen, Bergen, Norway; ^2^National Institute of Occupational Health, Oslo, Norway

**Keywords:** work stressors, role conflict, individual dispositions, trait anger, trait anxiety, affect, workplace bullying

## Abstract

Stressors in the work environment and individual dispositions among targets have been established separately as antecedents and risk factors of workplace bullying. However, few studies have examined these stressors in conjunction in order to determine personal dispositions among targets as possible moderators in the work stressor–bullying relationship. The aim of the present study was to examine multiple types of dispositional affect among targets as potential moderators in the relationship between role conflict and exposure to bullying behaviors, employing two independent cross-sectional samples. The first sample comprised 462 employees from a Norwegian sea transport organization, where trait anger and trait anxiety were included moderators. The second sample was a nationwide probability sample of the Norwegian working population and comprised 1,608 employees randomly drawn from The Norwegian Central Employee Register, where positive and negative affect were included moderators. The results showed that trait anger, trait anxiety, and negative affect strengthened the positive relationship between role conflict and reports of bullying behaviors. Positive affect did not moderate this relationship. We conclude that the association between role conflict and bullying is particularly strong for those scoring high on trait anger, trait anxiety, and negative affect.

## Introduction

More than 20 years of research has documented workplace bullying as a prevalent and detrimental stressor in most contemporary organizations with antecedents and risk factors at many levels of analyses. Workplace bullying is defined as an employee’s systematic exposure to unwanted behaviors from one or more coworkers, where the target has difficulties defending her- or himself against these negative acts (Olweus, 1991; Einarsen et al., 2011). Bullying is not an *either-or* phenomenon but rather a gradually escalating process where the person confronted ends up in an inferior position with little recourse to retaliate in kind (see Einarsen and Skogstad, 1996; Einarsen et al., 2011). While the causes of bullying may be multifold and difficult to establish, the two prevailing perspectives on the occurrence of bullying are: (1) the “Individual Disposition Hypothesis,” and (2) the “Work Environment Hypothesis” (Salin and Hoel, 2011; Zapf and Einarsen, 2011; Nielsen et al., 2017). The “Individual Disposition Hypothesis” highlights individual characteristics among either targets and/or perpetrators, such as personality traits, as potential precursors of bullying and claims that specific characteristics, or combinations of characteristics, increase the risk of becoming a target or a perpetrator of bullying (Zapf and Einarsen, 2011; Nielsen and Knardahl, 2015). In support of psychosocial work factors as causes of bullying, the “Work Environment Hypothesis” (Einarsen et al., 1994; Leymann, 1996; Baillien et al., 2009) states that negative and poorly organized work environments foster bullying through creating distress and conflicts among the employees. As such, role conflict has been shown to be a particularly strong predictor of bullying across industries (Bowling and Beehr, 2006; Reknes et al., 2014).

While there is general agreement within the research field that both personality dispositions and psychosocial work factors at work can act as antecedents of bullying, there are few studies examining these factors in conjunction. In addition, most studies have only investigated direct effects and there is consequently a shortage of studies on the moderating factors that determine when and for whom a given variable functions as an antecedent of workplace bullying (Francioli et al., 2016; Van den Brande et al., 2016). Personal factors affect the way individuals typically appraise external stimuli, react to them, and cope with them (Lazarus and Folkman, 1984). As such personal dispositions may also influence the way employees perceive and deal with their psychosocial work environment, as well as the outcomes that result from this experience (Francioli et al., 2016; Van den Brande et al., 2016). To empirically test the potential moderating effect of individual dispositions on the associations between work environment factors and exposure to bullying behaviors, we examined the importance of dispositional affect in the role conflict–bullying relationship, using two independent samples. In this “dispositional affect” represents an umbrella term for more or less stable affective traits and tendencies, as measured by trait anxiety and trait anger, as well as negative and positive affect, respectively. Empirically, these factors has been shown the be the best documented individual risk factors for exposure to workplace bullying, according to a recent meta-analysis (Nielsen et al., 2017). These factors also follows theoretically from the victim-precipitation theory, stating that individual factors that may make you vulnerable and weak or provoking and aggressive may elicit aggression in others turning the focal person into a target (Elias, 1986).

Role conflict refers to a situation where two or more perceived expectations in the work environment are not mutually compatible in that there is a mismatch in demands placed upon an employee such that compliance with both would be difficult (Beehr, 1995). Role stressors, and in particular role conflict, have been established as one of the work stressors most consistently related to reports of exposure to workplace bullying. This relationship has been established in both cross-sectional (Notelaers et al., 2010) and longitudinal studies (Balducci et al., 2012; Reknes et al., 2014), as well as meta-analyses and systematic reviews (Bowling and Beehr, 2006; Van den Brande et al., 2016). Findings show that victims of bullying, as well as bullies and bystanders, report the presence of role conflicts, lack of information and unclear tasks in their work environment (Agervold and Mikkelsen, 2004; Hauge et al., 2007). As such, people subjected to role conflicts may see the situation as a hindrance, because of a perceived threat toward personal development and goal attainment (Cavanaugh et al., 2000; Podsakoff et al., 2007). The increased risk of being exposed to bullying may thus follow from the target’s reactions to the perceived role conflict, as described in the “Victim Precipitation Theory” (Elias, 1986) discussed below.

Although role conflict has been established as a significant predictor of workplace bullying in studies of both targets (Einarsen et al., 1994), perpetrators (Hauge et al., 2011b), and departments (Hauge et al., 2011a) it is unlikely that all employees will experience and react to role conflicts in a similar manner. Actually, there has been widespread agreement among personality psychologists that behavior is jointly determined by the interaction of both person and situation variables (Diener et al., 1984). To fully understand the impact of role conflicts on exposure to workplace bullying, it is therefore necessary to also consider the impact of individual dispositions among those involved, in our case target dispositions. Supporting this view, several studies have shown that personality characteristics among targets increase the risk of being exposed to bullying from others (Podsiadly and Gamian-Wilk, 2016; Nielsen et al., 2017). Illustrative of this, negative affectivity (Bowling et al., 2010), trait anxiety, and trait anger (Vie et al., 2010), have all been related to employees who report exposure to bullying at work. However, in light of the great interest in personality factors as possible antecedents of bullying, it is noteworthy that studies looking at the role of personal dispositions as moderators in the work stressor–bullying relationship are scarce. The present study will address this general gap in the literature by looking at predispositions of targets as a moderator between role conflict and one’s exposure to workplace bullying. The neglect of such personal dispositions as possible moderators in this otherwise well-documented relationship, is therefore a major limitation in current research. One exceptions though, is the study by Balducci et al. (2011) investigating the moderating effect of neuroticism in the relationship between job demands (role conflict and work load) and exposure to bullying. Despite expectations of a relationship this study did not demonstrate any clear associations between neuroticism and bullying. Yet, this study looked at a more vague and complex demand factor and did not focus strictly on role conflict, which again is found to be one of the most important psychosocial predictors of workplace bullying across organizational settings and industries (Einarsen et al., 1994; Hauge et al., 2007).

Theoretically, the inclusion of these personal dispositions in the work stressor–bullying relationship, may be justified by the “Victim Precipitation Theory” (Elias, 1986), arguing that the victim may possess or exhibit certain characteristics or behaviors and reactions that may provoke or elicit victimization from others (Tepper et al., 2006). According to this theory there are two victim archetypes; the vulnerable victim who experience bullying because he/she appears weak, and the provocative victim who experience bullying because he/she has provoked the perpetrator (Olweus, 1978; Aquino and Lamertz, 2004; Samnani and Singh, 2016). Such provocative behavior may be founded in personality dispositions or may arise from stressors in the work environment, in our case theorized to arise from experiences of role stress (see Einarsen et al., 1994). We then propose that certain individual dispositions may further exacerbate targets’ experience and reactions to work related role stress, which again may further provoke and elicit negative behaviors in others, in our case as seen in targets’ report of being exposed to elevated levels of bullying behaviors while at work. Based on their tendency to react in certain ways, those high on negative affect, being the tendency to experience negative feelings as anger, fear, and sadness (Watson and Clark, 1984), will have an increased risk of exposure to such negative behaviors from others due to their expressed negativity related to their experiences of stressors at work (Watson and Clark, 1984; Fox and Spector, 1999). The same goes for those high on trait anxiety, characterized as the tendency to respond with anxiety to perceived stress in the environment (Spielberger, 1983), and trait anger, being sensitive to criticism and negative evaluation by others (Spielberger, 1996). As a result, people high on these types of dispositional affect may be more likely to interpret ambiguous information or conflicting expectations arising from role stress as threatening, even in seemingly innocent situations, and as a consequence, provoke more negative reactions from others, (Taylor and Kluemper, 2012), on our case as seen in their exposure to acts of workplace bullying. Hence, they may have a real risk of more exposure, as their frustration rooted in their feelings of role conflict may be interpreted by others as provoking or otherwise annoying (Samnani and Singh, 2016). On top of this, employees high on negative affect may also perceive more exposure to negative behaviors, but then more directly as a consequence of their dispositions. Yet, what we test in this study is that negative affective states and traits will strengthen the relationship between perceived role conflict and one’s exposure to workplace bullying. Positive affect, on the other hand, may act as a buffer in this relationship, as positive emotions are related to engagement and a more positive view of one’s surroundings (Watson et al., 1988; Weiss and Kurek, 2003). As such, people scoring high on positive affect may perceive stress and work demands as less demanding and thereby cope more easily with stressful events (De Lange et al., 2005), hence being in less risk of being retaliated against when under stress.

The relationship between role conflict and bullying is well-known in the literature. Yet, far more research is needed to shed light on the possible role of dispositional affect as a moderator in the role conflict–bullying relationship. In order to investigate the assumption that both positive and negative affective states and traits among targets may moderate the relationship between role conflict and exposure to bullying we set out to test the following hypotheses in a two-sample study employing different concepts and measures of affective dispositions, as well as of role-conflict:

H1. Role conflict is positively associated with exposure to bullying behaviors (to be tested in samples 1 and 2).

H2. The relationship between role conflict and exposure to bullying behaviors is stronger for those targets high on *trait anger*, as opposed to those with a low score (sample 1).

H3. The relationship between role conflict and exposure to bullying behaviors is stronger for those targets high on *trait anxiety*, as opposed to those with a low score (sample 1).

H4. The relationship between role conflict and exposure to bullying behaviors is stronger for those targets high on *negative affect*, as opposed to those with a low score (sample 2).

H5. The relationship between role conflict and exposure to bullying behaviors is weaker for those targets high on *positive affect*, as opposed to those with a low score (sample 2).

## Materials and Methods

### Sample 1

#### Design and Procedure

The first sample was based on data from a questionnaire survey sent by Norwegian Postal Services during the autumn 2007 and the winter 2008, to all 837 employees in a Norwegian sea transport organization. The respondents could either answer the questionnaire on paper, or use a login code printed on the questionnaire to answer the questionnaire online. Approximately 30% of the respondents used the online services. Altogether 462 respondents answered the questionnaire regardless of the chosen procedure, leaving us with a response rate of 55%.

#### Sample

The sample consisted of 246 officers and 216 crew members, where 82% (*n* = 379) were males and the mean age was 45 years (*SD* = 11.76), ranging from 17 to 66. The majority of the sample reported to be in a full time employment (93.2%). Fifty seven percent of the respondents were on daily working time arrangement.

#### Instruments

Five items from Rizzo et al. (1970) scale were used to measure role conflict, with response categories ranging from 1 = “*Very false*” to 7 = “*Very true.*” Cronbach’s alpha for this scale was 0.81.

The State-Trait Anxiety Inventory (STAI) and the State-Trait Anger Expression Inventory (STAXI) were used to measure trait anger and trait anxiety (Spielberger, 1983, 1996). Trait anxiety was measured with 20 items (e.g., *“I feel nervous and restless”*) with response categories ranging from 1 = “*Not at all*” to 4 = “*Very much.*” Trait anger was measured with 12 items (e.g., “*I get angry when other people’s mistakes go beyond me”)* with response categories ranging from 1 = “*Almost never*” to 4 = “*Almost always.*” The scales had a Cronbach’s alpha value at 0.88 and 0.75, respectively.

The short version of the Negative Acts Questionnaire (S-NAQ: Notelaers and Einarsen, 2008; Einarsen et al., 2009) was used to measure exposure to bullying behaviors while at work, yet with no reference to the phrase bullying. The respondents were asked how often they had been exposed to nine negative behaviors during the last 6 months, with response categories ranging from 1 = “*Never*” to 5 = “*About daily.*” The S-NAQ scale had a Cronbach’s alpha value at 0.88.

### Sample 2

#### Design and Procedure

The second sample was a probability sample of the Norwegian working force, drawn from The Norwegian Central Employee Register by Statistics Norway (SSB; the governmental agency for public statistic in Norway). The Norwegian Central Employee Register is the official register of all Norwegian employees, as reported by employers. The sampling criteria were adults between 18 and 60 years of age that were registered in the Central Employee Register as employed during the last 6 months, in a Norwegian enterprise with a staff of five or more and with a mean working hour of more than 15 h per week. Questionnaires were distributed through the Norwegian Postal Service during the spring 2015, to a random sample of 5,000 employees. A total of 1,608 questionnaires were satisfactory completed and included in this study (32% response rate).

#### Sample

Mean age in the sample was 45 (*SD* = 10.04) years with a range from 21 to 61. The sample consisted of slightly more women (52%) than men (48%). Altogether 9.4% had less than 11 years of education, 31% had between 11 and 13 years, 32% had between 14 and 17 years, while 27.8% had 18 years or more. This result reflects the high level of education in the Norwegian workforce. The majority of the sample reported to be in a full time (89.4%) or part time (6.6%) employment. About 3.6% were on a sick leave or occupational rehabilitation, whereas 0.5% reported to be disabled pensioners or retired. Eighty one percent of the respondents were on daily working time arrangement. Altogether 36% had a leadership position with personnel responsibilities; hence supervisors/managers are somewhat overrepresented in the sample.

#### Instruments

As in sample 1, the short Negative Acts Questionnaire (S-NAQ: Notelaers and Einarsen, 2008) was used to measure exposure to nine specific negative acts. The S-NAQ scale had a Cronbach’s alpha value of 0.86.

Role conflict was assessed with three items from the General Nordic Questionnaire for Psychological and Social Factors at Work (QPSNordic; Dallner et al., 2000). Answers were provided on a four-point scale ranging from 1 = “*Always*” to 4 = *“Never.”* Cronbach’s alpha was low (0.60), yet regarded as acceptable given that it only consisted of three items with moderate inter-correlations between the three items (0.39, 0.36, and 0.34, respectively).

Ten items from the Positive and Negative Affect Schedule X (PANAS-X: Watson and Clark, 1994) were used to assess experienced negative and positive emotions. With reference to five negative and five positive emotions, the respondents were asked to what extent they generally had felt this way during the last couple of weeks. Response categories are ranging from 1 = “*Not at all/very slightly”* to 5 = *“Very much.”* The internal consistency for the negative affect (NA) scale (Cronbach’s alpha = 0.75) and the positive affect (PA) scale (Cronbach’s alpha = 0.78), was satisfactory in the present study.

### Statistical Analysis

Statistical analyses were conducted with IBM SPSS 22.0. The level of significance was set to *p* < 0.05. For all measurement inventories, summary scales were calculated on the basis of a mean-score of their respective items. To explore the hypotheses about main and moderating effects, we conducted four independent hierarchical regression analyses, to test for linear associations between role conflict and exposure to bullying behaviors as well as the interactive effects of role conflict and trait anger/trait anxiety/negative affect/positive affect, respectively, with regard to bullying. The recommendations provided by Baron and Kenny (1986) were followed, and, in accordance with Aiken and West (1991), the predictor variables were centered prior to the two-way interaction analysis. The SPSS macro “Interaction and simple slopes test with two continuous variables” by Jason T. Newsom^1^ was used to generate the regression estimates, plots, and simple slopes analyses.

### Ethics Statement

The surveys included in this paper were approved by the Regional Committee for Medical Research Ethics, for Eastern and Western Norway (sample 2), and the Norwegian Social Science Data Services (NSD) (sample 1). Participation was voluntary, based on informed consent and withdrawal from the studies was allowed at any given time.

## Results

### Sample 1

Descriptives, reliability coefficients, and intercorrelations for all study variables in sample 1 are presented in Table 1. The intercorrelations showed that role conflict was positively associated with trait anger (*r* = 0.25; *p* < 0.001), trait anxiety (*r* = 0.28; *p* < 0.001), and exposure to bullying behavior (*r* = 0.44; *p* < 0.001). Also, trait anger (*r* = 0.31; *p* < 0.001) and trait anxiety (*r* = 0.36; *p* < 0.001) were positively associated with exposure to bullying behaviors.

**Table 1:** Means, standard deviations, inter-correlations (Pearson’s *r*), and Cronbach’s alpha’s (in bold along diagonal) for study variables in sample 1.

	Measure	*M*	*SD*	1	2	3	4
1.	Role conflict	3.19	1.33	**0.81**			
2.	Trait anger	1.49	0.32	0.25^∗∗∗^	**0.75**		
3.	Trait anxiety	1.57	0.38	0.28^∗∗∗^	0.32^∗∗∗^	**0.88**	
4.	Bullying behavior	1.29	0.43	0.44^∗∗∗^	0.31^∗∗∗^	0.36^∗∗∗^	**0.88**


*Level for significance: ^∗∗∗^*p* < 0.001.*

### Trait Anger as Moderator

#### Main and Interaction Effects

Findings from the multiple regression analysis of linear associations and interaction effects, including the variables role conflict, *trait anger* and bullying, are presented in Table 2. Both role conflict and trait anger yielded significant contributions to the variance (*F* = 58.57; *df* = 2; *p* < 0.001). The results showed that role conflict (β = 0.39; *p* < 0.001) had a significant linear effect on exposure to bullying behaviors, thus supporting H1. Also, trait anger was associated with the outcome variable (β = 0.17; *p* < 0.001), and hence the predictor variables together explained 21.4% of the variance in reported exposure to bullying (*R*^2^ = 0.214; *p* < 0.001). Furthermore, the interaction effect of role conflict and trait anger was significant (β = 0.21; *p* < 0.001), supporting H2. When adding the interaction term, the amount of variance increased significantly by 4.2% (*R*^2^ = 0.255; *p* < 0.001). The interaction model was significant (*F* = 49.19; *df* = 3; *p* < 0.001).

**Table 2:** Testing the moderator effect of trait anger in the relationship between role conflict (predictor) and exposure to bullying behaviors (outcome) using hierarchical multiple regression in sample 1.

	Predictor	*ΔR*^2^	β
Step 1		0.214	
	Role conflict		0.39^∗∗∗^
	Trait anger		0.17^∗∗∗^
Step 2		0.042	
	Role conflict		0.38^∗∗∗^
	Trait anger		0.15^∗∗∗^
	Role conflict^∗^trait anger		0.21^∗∗∗^
Total *R*^2^	0.255^∗∗∗^		
*N*	433		


*Level for significance: ^∗∗∗^*p* < 0.001.*

**FIGURE 1:**
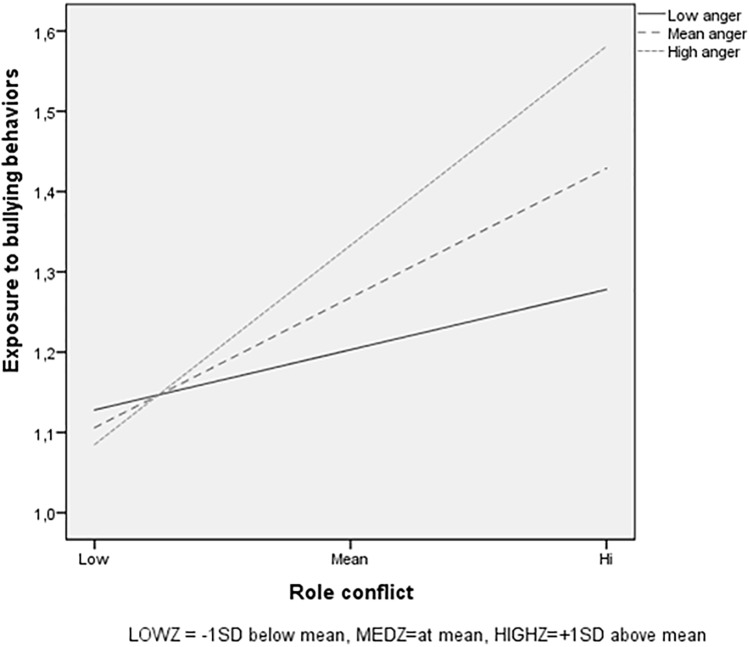
Trait anger as moderator in the relationship between role conflict and exposure to bullying behaviors, for low role conflict (1 *SD* below the mean), for medium role conflict (mean), and for high role conflict (1 *SD* above the mean).

To examine the form of the interaction, a graphical display was created, based on the recommendations by Cohen et al. (2003) and Frazier et al. (2004). Scores were plotted at the mean, low (1 *SD* below the mean) and high (1 *SD* above the mean) values on the predictor variables. The results show that when experiencing low levels of role conflict the likelihood of being exposed to bullying behaviors is low for all employees, regardless of their level of trait anger (Figure 1). Experiencing high levels of role conflict on the other hand is related to an increase in exposure to bullying behaviors for all groups. However, those with high scores on trait anger are more exposed than those with lower scores. Follow-up analyses of simple slopes revealed that higher levels of role conflict were more strongly related to exposure to bullying among respondents with mean (β = 0.37; *p* < 0.001) and high trait anger (β = 0.57; *p* < 0.001), as compared to for respondents with low levels of trait anger (β = 0.17; *p* < 0.05).

### Trait Anxiety as Moderator

#### Main and Interaction Effects

Findings from the multiple regression analysis of linear associations and interaction effects, including the variables role conflict, *trait anxiety* and exposure to bullying behaviors, are presented in Table 3. For the linear associations, the predictor variables explained 24.4% of the variance in bullying behaviors (*R*^2^ = 0.244; *p* < 0.01). Both role conflict (β = 0.36; *p* < 0.001) and trait anxiety (β = 0.25; *p* < 0.001) yielded significant contributions to the variance (*F* = 69.89; *df* = 2; *p* < 0.001). Furthermore, the interaction effect of role conflict and trait anxiety on exposure to bullying behaviors was significant (β = 0.23; *p* < 0.001), increasing the amount of explained variance by 5% (*R*^2^ = 0.295; *p* < 0.001). Hence, the interaction model was significant (*F* = 59.99; *df* = 3; *p* < 0.001), supporting H3.

**Table 3:** Testing the moderator effect of trait anxiety in the relationship between role conflict (predictor) and exposure to bullying behaviors (outcome) using hierarchical multiple regression in sample 1.

	Predictor	*ΔR*^2^	β
Step 1		0.244	
	Role conflict		0.36^∗∗∗^
	Trait anxiety		0.25^∗∗∗^
Step 2		0.050	
	Role conflict		0.35^∗∗∗^
	Trait anxiety		0.20^∗∗∗^
	Role conflict^∗^trait anxiety		0.23^∗∗∗^
Total *R*^2^	0.295^∗∗∗^		
N	434		


*Level for significance: ^∗∗∗^*p* < 0.001.*

**FIGURE 2:**
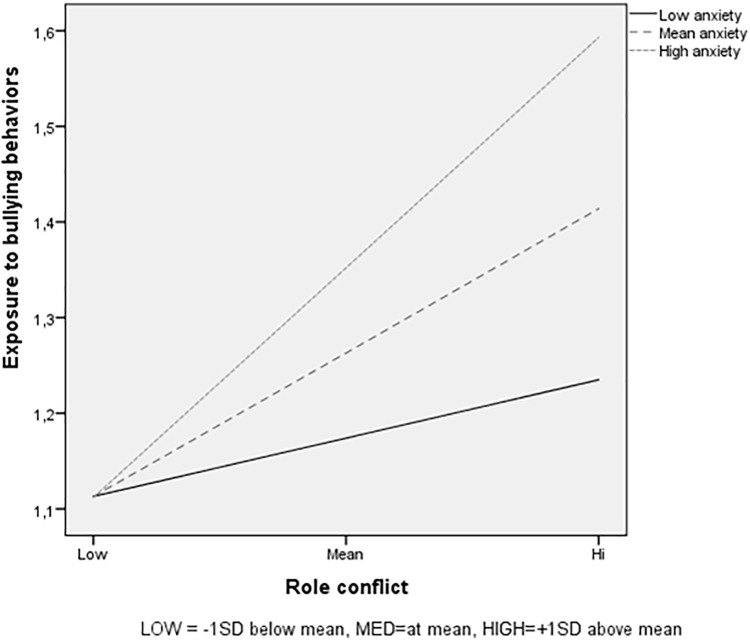
Trait anxiety as moderator in the relationship between role conflict and exposure to bullying behaviors, for low role conflict (1 *SD* below the mean), for medium role conflict (mean), and for high role conflict (1 *SD* above the mean).

The interaction effect is graphically displayed in Figure 2. The results show again that when experiencing low levels of role conflict the likelihood of being exposed to bullying behaviors is small for all employees, here regardless of their level of trait anxiety. Experiencing high levels of role conflict on the other hand is related to an increase in exposure to bullying behaviors for all, and especially for those with a high score on trait anxiety. Follow-up analyses of simple slopes revealed that higher levels of role conflict were more strongly related to higher exposure to bullying among respondents with mean (β = 0.35; *p* < 0.001) and high trait anxiety (β = 0.56; *p* < 0.001), than for respondents with low levels of trait anxiety (β = 0.14; *p* < 0.05).

To sum up, the results of our hypotheses show that trait anxiety and trait anger act as enhancement factors in the relationship between role conflict and exposure to bullying behaviors. For those with higher scores on trait anxiety and/or trait anger the level of exposure to bullying behaviors increases dramatically when experiencing high levels of role conflict at work as opposed to those with low trait anger/anxiety, who hardly have any increased risk of experiencing bullying under high role conflict.

### Sample 2

Descriptives, reliability coefficients, and intercorrelations for all study variables in sample 2 are presented in Table 4. The intercorrelations showed that role conflict was positively associated with both negative affect (NA) (*r* = 0.30; *p* < 0.001) and exposure to bullying behavior (*r* = 0.40; *p* < 0.001), while negatively related to positive affect (PA) (*r* = -0.10; *p* < 0.001). Furthermore, NA was positively associated with bullying behavior (*r* = 0.43; *p* < 0.001), while PA was negatively related to bullying behavior (*r* = -0.11; *p* < 0.001).

**Table 4:** Means, standard deviations, inter-correlations (Pearson’s *r*), and Cronbach’s alpha’s (in bold along diagonal) for study variables in sample 2.

	Measure	*M*	*SD*	1	2	3	4
1.	Role conflict	1.81	0.43	**0.60**			
2.	Negative affect	1.37	0.51	0.30^∗∗∗^	**0.75**		
3.	Positive affect	3.59	0.70	-0.10^∗∗∗^	-0.11^∗∗∗^	**0.78**	
4.	Bullying behavior	1.19	0.34	0.40^∗∗∗^	0.43^∗∗∗^	-0.11^∗∗∗^	**0.86**


*Level for significance: ^∗∗∗^*p* < 0.001.*

### Negative Affect as Moderator

#### Main and Interaction Effects

Findings from the multiple regression analysis of linear associations and interaction effects, including role conflict, NA, and exposure to bullying behaviors are presented in Table 5. For the linear association, the predictor variables explained 27% of the variance in bullying (*R*^2^ = 0.269; *p* < 0.001). Both role conflict (β = 0.30; *p* < 0.001) and NA (β = 0.34; *p* < 0.001) yielded significant contributions to the variance (*F* = 290.40; *df* = *2*; *p* < 0.001). Hence H1 was also supported in this sample. When adding the interaction term to the regression analysis, the amount of explained variance increased significantly by 5.2% (*R*^2^ = 0.321; *p* < 0.01). As displayed in Table 5, the interaction term made a significant contribution to the explained variance in reported exposure to bullying (β = 0.25; *p* < 0.001). The magnitude of the associations between the predictor variables and bullying behaviors was somewhat attenuated, but still significant, after including the interaction term. The interaction model was significant (*F* = 248.17; *df* = 3; *p* < 0.001), supporting H4.

**Table 5:** Testing the moderator effect of negative affect in the relationship between role conflict (predictor) and exposure to bullying behaviors (outcome) using hierarchical multiple regression in sample 2.

	Predictor	*ΔR*^2^	β
Step 1		0.269	
	Role conflict		0.30^∗∗∗^
	Negative affect		0.34^∗∗∗^
Step 2		0.052	
	Role conflict		0.27^∗∗∗^
	Negative affect		0.26^∗∗∗^
	Role conflict^∗^negative affect		0.25^∗∗∗^
Total *R*^2^	0.321^∗∗∗^		
*N*	1580		


*Level for significance: ^∗∗∗^*p* < 0.001.*

**FIGURE 3:**
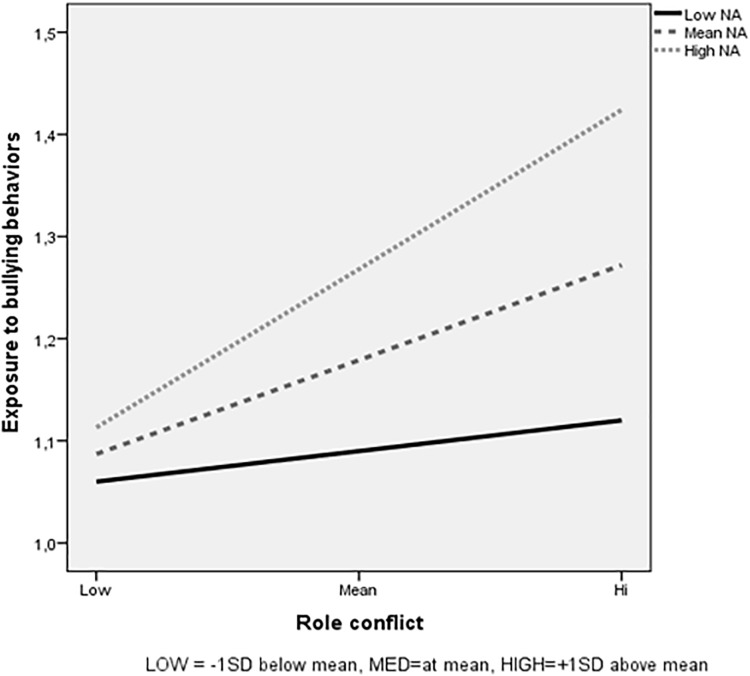
Negative affect (NA) as moderator in the relationship between role conflict and exposure to bullying behaviors, for low role conflict (1 *SD* below the mean), for medium role conflict (mean), and for high role conflict (1 *SD* above the mean).

As shown in Figure 3, the results indicate a stronger relationship between role conflict and bullying behavior for respondents with mean and high NA than for the low NA group. Follow-up analyses of simple slopes revealed that higher levels of role conflict were related to significantly higher exposure to bullying behaviors among respondents with mean (β = 0.27; *p* < 0.001) and high NA (β = 0.45; *p* < 0.001), but not for respondents with low NA (β = 0.09; *p* > 0.05).

### Positive Affect as Moderator

#### Main and Interaction Effects

Findings from the multiple regression analysis of linear associations and interaction effects, including role conflict, PA, and exposure to bullying behaviors are presented in Table 6. For the linear association, the predictor variables explained 16.6% of the variance in bullying behavior (*R*^2^ = 0.166; *p* < 0.001). Both role conflict (β = 0.39; *p* < 0.001) and PA (β = -0.07; *p* < 0.001) yielded significant contributions to the variance. However, the results showed that PA did not moderate the association between role conflict and exposure to bullying behaviors. Hence, H5 was not supported.

**Table 6:** Testing the moderator effect of positive affect in the relationship between role conflict (predictor) and exposure to bullying behavior (outcome) using hierarchical multiple regression in sample 2.

	Predictor	*ΔR*^2^	β
Step 1		0.166	
	Role conflict		0.39^∗∗∗^
	Positive affect		-0.07^∗∗^
Step 2		0.002	
	Role conflict		0.27^∗∗∗^
	Positive affect		-0.07^∗∗^
	Role conflict^∗^positive affect		-0.04^ns^
Total *R*^2^	0.168		
*N*	1580		


Level for significance: ^∗∗∗^*p* < 0.001; ^∗∗^*p* < 0.01; ns, not significant.

## Discussion

The aim of the present study was to investigate the relationship between role conflict, dispositional affect in targets, and exposure to bullying behaviors, particularly looking at the moderating role of dispositional affect in the otherwise well documented role conflict–bullying relationship. Based on theory and earlier empirical data, role conflict was expected to be positively related to exposure to bullying behaviors (H1). Furthermore, four types of dispositional affect in targets; trait anger (H2), trait anxiety (H3), negative affect (NA) (H4), and positive affect (PA) (H5), were suggested as possible moderators in the relationship between role conflict and exposure to bullying behaviors. All hypotheses were supported, except for H5, and the results thus indicate that the relationship between role conflict and reported exposure to workplace bullying is particularly strong for employees high on negative affective states and traits.

### Role Conflict and Workplace Bullying

The results in this paper show, as expected, a positive relationship between role conflict and exposure to bullying behaviors at work, supporting H1. This finding leans on previous research (Jennifer et al., 2003; Hauge et al., 2007, 2011a), as well as the “Work Environment Hypothesis,” indicating that conflicting expectations in the work environment may provoke bullying to occur (Leymann, 1996; Bowling and Beehr, 2006), both through mechanisms described in the “Frustration-Aggression Hypothesis” (Berkowitz, 1989) when focusing on the perpetrator, and in the “Victim Precipitation Theory” (Elias, 1986) when looking at respondents in the role of targets as was the case in the present study.

In line with Van de Vliert (1998) conflict model the direction in which a conflict develops is dependent on how the parties choose to communicate during the conflict escalation. As role conflict is referred to as a situation with poor communication between role senders (Jex, 1998), it may be easy for the person who experience the role conflict to act with frustration against the role senders, which then may intensify the conflict by mechanisms of counter attacking and venting their own frustration (see also Balducci et al., 2012). Hence, the conflict’s content may change from being purely task related in the start to gradually becoming more related to the included parties’ personal characteristics and involving more and more aggressive outlets (Baillien et al., 2009), with perceived or real exposure to bullying behaviors as a result. This may be particular so when the role senders are known to the focal person, because the cause of the role conflict can then more easily be attributed to their behaviors and intentions (Einarsen et al., 1994). Illustrative of this, some scholars have suggested that the relationship between role conflict and workplace bullying can be explained by the targets reactions to the conflict, assuming that he or she acts in a way others find irritating, hence increasing his or her risk of being bullied (Neuman and Baron, 2003; Bowling and Beehr, 2006; Notelaers et al., 2010), a proposition in line with the “Victim Precipitation Theory” by Elias (1986).

### The Moderating Role of Dispositional Affect

People with positive emotions tend to have more positive experiences of events or a more rosy perception of reality than do others (Weiss and Kurek, 2003). In contrast, people characterized by negative emotions and negative affect tend to have more negative, or gloomy, perceptions of events. Based on this view we assumed that positive affect could act as a protective buffer in the role conflict–bullying relationship, whereas disposition such as negative affect, trait anger and trait anxiety would act as vulnerability and enhancement factors. Contrary to our expectations, positive affect did not have an impact on the role conflict–bullying relationship, indicating that when experiencing role conflict all employees are in risk of reporting exposure to bullying behaviors, irrespective of their disposition to experience and retain a positive affect state. On the other hand, trait anger, trait anxiety, and negative affect strengthened the relationship between role conflict and exposure to workplace bullying, where those scoring high, or even just having a mean score on these affect dispositions, showed a stronger relationship as compared to those scoring low.

The fact that PA did not play a role in the relationship between role conflict and bullying may be surprising at first, as those high on PA is supposed to perceive stress and work demands as less demanding (De Lange et al., 2005), and as a result may report less victimization. A possible explanation may, however, be found in the assumption that role conflict is problematic for all involved, even though “bad may be stronger than good.” In their review, Baumeister et al. (2001) concluded that NA and emotional stress have more impact than positive emotions and affective states. They explain this in relation to the level of cognitive processing, where negative information receives more processing and contributes more strongly to the final impression of the situation (Baumeister et al., 2001). Those high on NA, referred to as the tendency to be upset and distressed, are shown to have a more negative view of others and self (Watson and Clark, 1984; Djurkovic et al., 2004). Also, NA has been related to poor coping, health complaints and self-reported stress (Watson et al., 1988). Similarly, neurotic individuals, with the tendency to experience negative emotions (Costa and McCrae, 1992), may be more prone to perceiving others behaviors as insults and threats (Taylor and Kluemper, 2012), thus reporting more exposure to bullying behaviors from the same people. Hence, one’s tendency to experience negative states and traits in conflict situations is thought to have severe outcomes, as supported by the results presented in this study. However, at low levels of role conflict the bullying exposure level was the same for all groups, regardless of their negative or aggressive feelings and behaviors. Hence, it seems like it is the combination of high role conflict and high scores on these negative affect dispositions that relates to bullying exposure, rather than the affect dispositions in themselves.

The present study adds new and important knowledge to the understanding of how personal dispositions impact the work stress-bullying relationship, as few studies have investigated these mechanisms earlier (for exeptions, see: Balducci et al., 2011; Francioli et al., 2016). Theoretically, the results may be explained in line with the “Victim Precipitation Theory,” which offers insight into who is likely to become a victim of bullying by presenting a vulnerable and a provocative victim approach (Elias, 1986; Samnani and Singh, 2016). It may also be that employees high on negative affect dispositions will be predisposed to perceive others’ behaviors as negative regardless of the objective nature of the behavior, evoking negative emotions that when displayed may increase their risk of subsequent victimization. It may also be the case that, the negative response to role conflict is stronger among these employees leading them to perceive the behaviors and responses of others as being more hostile (Spector et al., 2000).

### Methodological Issues

The use of two independent samples, as well as the use of varied measures and operationalization of the investigated concepts (i.e., role conflict and dispositional affect), is a particular strength of the present study. The use of cross-sectional data, however, decreases the possibility to draw causal explanations for the findings. To say something about cause and effect, longitudinal studies are needed (see: Hauge et al., 2011b; Nielsen and Knardahl, 2015). However, even when using cross-sectional data, moderation models are casual models by nature due to the underlying theories suggesting directional inferences which are intrinsically causal (Wu and Zumbo, 2008).

The variance explained by the moderators in this study, ranging from 4.2 to 5.2%, may seem small, at least at first glimpse. However, such moderator effects are in general difficult to detect, and one cannot expect large contributions to the variance from such interactions (McClelland and Judd, 1993). In a study on the interaction between self-efficacy and exposure to workplace bullying, the interaction explained 2% of the variance in psychological health complaints (Mikkelsen and Einarsen, 2002). A study investigating the interaction between laissez-faire leadership and decision authority with regard to workplace bullying, added some 2% to the overall variance (Hauge et al., 2007). Taking this into consideration, the effect sizes in our paper are in fact quite large, both when we compare our results with other studies on moderation (e.g., Tepper et al., 2009) and when looking at the plots of the results showing gross differences in risk of exposure when facing role-conflict between those low and those high on these individual dispositions.

### Implications

The clear-cut results from these two samples have theoretical, methodological and applied implications. First of all, they show the importance of including moderating factors when examining antecedents of workplace bullying, be it in empirical studies as well as in future theories and models of antecedents of workplace bullying. As we found significant interaction effects of different forms of dispositional affect on the relationship between role conflict and exposure to bullying behaviors, our findings suggest that the potential impact of role conflict may vary between individuals and by just examining direct associations between the variables one may underestimate the actual impact of the predictor. As for the practice field, the results in the present study suggest that management interventions should aim at reducing conflicting roles at work in order to avoid escalating conflicts and bullying to occur in the workplace, for instance by offering conflict management training and proper communication channels between superiors and subordinates. It is also important for managers to recognize that there seem to be gross individual differences in how such role conflicts are handled, and that personal characteristics in terms of anxiety and aggression seem to escalate the chance of being bullied, or at least reporting being exposed to bullying behaviors, when facing conflicting demands at work. Yet, another important applied implication from this research is that even though personal dispositions may be risk factors for bullying, they are particularly so in situations with a problematic working environment. When no role conflict existed, people high in negative affect did not report more exposure to bullying behaviors. Hence, the main interventions to prevent bullying need to be in relation to the working environment. For both managers and HR-personnel, this is important knowledge. In addition, organizations always need to put in place proper policies and procedures in order to build up a strong organizational infrastructure to handle all individual complaints of bullying in a proper manner.

## Conclusion

The results in the present study indicate that dispositional affect moderates the well-documented relationship between role conflict and workplace bullying, in that the association between role conflict and exposure to bullying behaviors is stronger for those scoring high on trait anger, trait anxiety and negative affect, compared to those with a low score on these affect dispositions. This may be due to the target’s provocative behavior resulting from elevated levels of stress and frustration, or the fact that he or she is perceived as an easy target due to a the tendency to show strong emotions of anger or anxiety. It may also be that employees scoring high on more negative affect dispositions perceive others’ behaviors as insulting regardless of the behaviors’ objective nature, and as a result report more exposure to bullying behaviors. Yet, role conflict is associated with increased risk of becoming a target of bullying irrespective of one’s tendency to show positive emotions. The study opens a wide avenue of further person-environment studies of antecedents of workplace bullying, as well as opening avenues of preventive measures directed toward both the general working environment and staff in general, as well as measures tailor-made for those in particular risk due to individual pre-dispositions.

## Author Contributions

All authors have been responsible for the study’s concept and design. Also, all authors have been actively involved in the writing process, and are collectively responsible for the final completion of the manuscript.

## Conflict of Interest Statement

The authors declare that the research was conducted in the absence of any commercial or financial relationships that could be construed as a potential conflict of interest.

## Footnotes

1 http://web.pdx.edu/~newsomj/
